# Toward AI-Assisted Greener Chiral HPLC: Predicting Efficient Enantioseparation–Mobile Phase (EES–MP) Profiles for MP Selection—A Lux Cellulose-1 Case Study

**DOI:** 10.1021/acs.analchem.5c06117

**Published:** 2025-12-22

**Authors:** Carlos Pardo-Cortina, Laura Escuder-Gilabert, María José Medina-Hernández, Salvador Sagrado, Yolanda Martín-Biosca

**Affiliations:** † Departamento de Química Analítica, 16781 Universitat de València, E-46100 Burjassot, Valencia, Spain; ‡ Instituto Interuniversitario de Investigación de Reconocimiento Molecular y DesarrolloTecnológico (IDM), Universitat Politècnica de València, Universitat de València, E-46100 Burjassot, Valencia, Spain

## Abstract

Chiral HPLC method development still relies heavily on trial-and-error screening. We introduce the Efficient Enantioseparation (EES) parameter—a single metric integrating resolution (Rs) and retention (k)—to move from point predictions to full mobile-phase (MP) profile modeling. Using EES as the response, we trained multiple artificial neural networks (ANNs) on 62 variables (molecular descriptors) and 76 objects (structurally diverse neutral and basic compounds chromatographed on a Lux Cellulose-1 column under aqueous–acetonitrile conditions at nine MP compositions). ANNs were optimized with a chaotic competitive-learning optimizer (CCLNNA), then ranked/selected and combined into a consensus model to enhance robustness and limit overfitting. The ANN-consensus model accurately reproduces full EES–MP profiles (*R* ^2^ > 0.9) with lower error dispersion, enabling prospective feasibility checks and single-shot selection of high-EES mobile-phase compositions. External tests on fluoxetine and lormetazepam confirmed prospective utility by anticipating separability at one or more MPs (nominating the MP with maximal EES) or nonseparability across the explored MP range. To our knowledge, this work provides the first proof-of-concept for in silico prediction of full EES–MP profiles in chiral HPLC, enabling intelligent MP selection. Rather than a definitive model, this work evaluates the potential of the strategy: consensus stabilizes learning with limited data and offers greener, actionable guidance that can reduce experiments, reagent consumption, and development time. The framework is extensible to broader chemotypes and stationary and mobile phases; larger data sets could further generalize EES-profile prediction and support intelligent MP selection in sustainable chiral HPLC.

## Graphical abstract

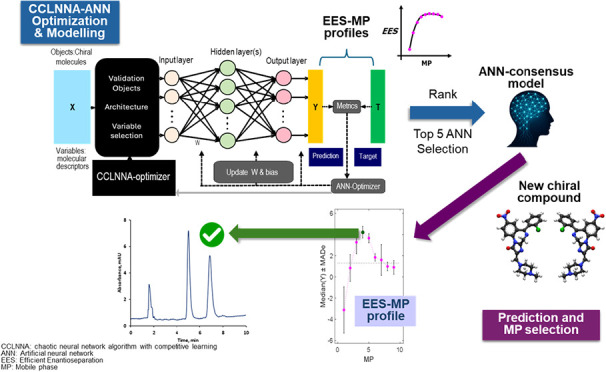

## Introduction

Chirality is a fundamental concept in analytical, clinical, and biological sciences, with profound implications for the activity, safety, and fate of bioactive compounds.^1^ A large proportion of naturally occurring substances are chiral,^2,3^ and their enantiomers frequently display distinct pharmacological, toxicological, and environmental behaviors due to the enantioselective nature of biological systems. As a result, the accurate separation and identification of enantiomers is of central importance, particularly in pharmaceutical research and development.^3–5^

High-performance liquid chromatography (HPLC) with chiral stationary phases (CSPs), especially polysaccharide-based materials such as cellulose and amylose, is widely regarded as the most versatile and selective approach for enantioseparation.^6–9^ However, the subtle free energy differences between diastereomeric complexes formed by enantiomers and chiral selectors often complicate method development.^5,10^ In practice, optimization has traditionally relied on trial-and-error strategies that are time-consuming, resource-intensive, and environmentally unsustainable (incompatible with green-chemistry principles and UN Sustainable Development Goals).^4,11,12^

To overcome these limitations, a variety of predictive and computational approaches have been developed. Molecular dynamics simulations provide atomistic insights into enantioselective recognition and allow prediction of elution order and thermodynamic parameters.^10^ Statistical modeling, including quantitative structure–retention/property relationships (QSRR/QSPR), has been applied to correlate molecular descriptors with retention times or separation performance.^13–18^ Techniques such as principal component analysis (PCA), multiple linear regression (MLR), partial least-squares (PLS), and discriminant PLS (DPLS) have also been used to estimate retention factors and predict enantioresolution outcomes.^16–20^

More recently, artificial intelligence (AI) has been increasingly integrated into chiral separations. Machine learning models, including random forests, artificial neural networks (ANNs), and graph neural networks (GNNs), have been used to predict CSP enantioselectivity, resolution, and elution order by combining molecular descriptors with experimental parameters.^14,16,21,22^ Deep learning strategies have shown superior performance compared with linear models in predicting chromatographic retention,^23,24^ and novel three-dimensional (3D) molecular representations have enhanced CSP selection compared with one-dimensional (1D) or two-dimensional (2D) approaches.^25^ In parallel, optimization algorithms such as genetic algorithms (GA) and neural network–based metaheuristics have been combined with chemometric methods to refine retention predictions,^13^ while more recent algorithms (e.g., chaotic competitive learning neural networks) have exhibited competitive search efficiency.^26^

In previous work, we introduced an intelligent recommendation system (IRS) based on optimized ANN ensembles, which recommended the most suitable CSP/mobile phase (MP) combinations (among 14 available options) for enantioresolution. Trained on 56 structural descriptors and optimized via a customized metaheuristic (CCLNNA) with a fit-for-purpose objective function targeting the recommendation score, the system achieved excellent recommendation performance.^12^ Building on this foundation, the present study proposes a novel AI-based approach to predict the optimal mobile phase composition (specifically the proportion of hydro-organic mixtures) for the enantioseparation of chiral compounds on given CSP/MP systems. We introduce the Efficient Enantioseparation (EES) parameter as a quantitative measure of separation efficiency and develop ANN-consensus models to predict full EES–MP profiles across diverse compounds. The objectives of this study are to (i) define and implement the EES metric, (ii) optimize ANN models with selected structural (input) variables employing a prediction-oriented CCLNNA optimizer, (iii) construct an ANN- consensus model from top-performing ANNs to yield robust EES–MP predictions, and (iv) test the model applicability for external compounds including uncertainty estimates. To our knowledge, this work represents the first proof-of-concept for in silico prediction of full EES–MP profiles, enabling intelligent MP selection in chiral HPLC separations.

## Experimental Section

### Data Set: Structural Variables and Chromatographic Measurements

To develop and evaluate the ANN-consensus model, we employed experimental chromatographic data and structural variables for 78 structurally diverse basic and neutral chiral compounds encompassing 20 pharmacological and agrochemical classes (see Supporting Information and Table S1). The predictor matrix (X-matrix) initially included 62 variables (see Table S2), 56 of which had been previously used.^27^ Six additional variables were introduced in this study: x8, x32, x37, x49, x50, and x51.

No explicit enantiomer-discrimination descriptors (i.e., with different numerical values for R and S enantiomers) were used. Instead, the data set explicitly incorporates information governing chiral recognition through chiral center-specific topological descriptors. Extending our previous methodology,^27^ variables x1–x7 characterize the immediate environment of the asymmetric carbon, quantifying substituents such as aromatic rings, heterocycles, and aliphatic chains attached to the chiral center. Additionally, this work introduces a new descriptor, x8 (number of N, O, or S atoms bonded to the chiral carbon), to further refine the characterization of the chiral center’s electronic environment, alongside global descriptors describing the entire molecular topology.

Chromatographic data, including retention factors (*k*_1_ and *k*_2_, for the least- and most-retained enantiomer, respectively) and enantioresolution (Rs) values, were obtained on a Lux Cellulose-1 (Cell1) column (150 × 4.6 mm^2^, 5 μm; Phenomenex) using nine mobile phases (MPs). MPs were prepared by mixing 5 mM ammonium bicarbonate buffer (pH 8.0) and acetonitrile (ACN), with ACN contents of 30–98% (v/v). The MP indices correspond to the following ACN fractions (% v/v): MP 1, 30%; MP 2, 40%; MP 3, 50%; MP 4, 60%; MP 5, 70%; MP 6, 80%; MP 7, 90%; MP 8, 95%; MP 9, 98%.

The flow rate was set to 1.0 mL·min^–1^, the column temperature was maintained at 25 °C, the injection volume was 2 μL (100 μg·mL^–1^ solution), and the detection wavelength was 220 nm. Data for 53 compounds were taken from a previous study,^27^ while 25 additional compounds (see Table S1) were newly analyzed in this work, expanding the data set.

### Efficient Enantioseparation: Definition and Calculation

In this study, a novel chromatographic parameter, efficient enantioseparation (EES), was introduced and applied to evaluate enantioseparation performance. EES penalizes excessively long retention times, providing a more practical measure of separation efficiency. EES, which integrates Rs and *k*_2_, is defined as

1 $$\mathrm{EES}=\mathrm{Rs}-\mathrm{p\cdot}\max\left(0,k_{2}-k_{2}^{\lim}\right)$$

where *p* is a constant dimensionless penalty factor, and *k*_2_^lim^ is the maximum acceptable *k*_2_; both p and *k*_2_^lim^ should be fixed a priori by the analyst. By construction, EES equals Rs when *k*_2_ < *k*_2_^lim^. In this work, some practical rules to work with EES were applied: (i) *k*_2_ was truncated at 30 and Rs was capped at 6. (ii) Experimental *k*_2_ and Rs values were rounded to one decimal place. (iii) We set *k*_2_^lim^ = 20 and a practical (acceptable) enantioresolution threshold of Rs ≥ 1.3 ^28^ (iv) EES values below −1 were set to −1; thus, the practical EES range was [−1, 6]. The optimal p value was determined via simulation using computer-generated Rs and *k*_2_ values, selecting *p* to limit false positives—cases where EES ≥ 1.3 but *k*_2_ > *k*_2_^lim^ (=20). A value of *p* = 45 was chosen, as it yielded a negligible false-positive rate of approximately 0.02%. As an example, using MP 2 (40% ACN), donepezil (ID 74 in Table S1) has an experimental Rs = 3.1 (complete enantioresolution) but with a high *k*_2_ of 51.2 (impractical analysis time). By eq 1, and after applying the specified rules, EES is set to −1, thereby considering the mobile-phase condition unsuitable. This example underscores why EES—not Rs alone—should guide MP selection. The target response **T**-matrix contained EES values for each compound measured at nine MPs (EES-MP profiles; Figure S1).

### Software and Calculations

The CCLNNA-ANN process was described in a previous publication.^12^

Both **X-** and **T-**matrices were autoscaled and all MP profile predictions (**Y**) were back-transformed to the original EES units at the end of the CCLNNA-ANN process.

To limit overfitting while preserving representativeness, data were split into training (Tr, 66 compounds), validation (Va, 7), and internal testing (IT, 3) subsets. Training decisions were driven by Va, whereas IT did not influence training and conditioned only the CCLNNA process. The Tr/Va/IT assignment was consistent with a balanced distribution of EES values. Architectures with up to two hidden layers were explored: the first with *N*_1_ neurons (1 ≤ *N*_1_ ≤ 30) and the second with *N*_2_ neurons (0 ≤ *N*_2_ ≤ 30) with *N*_2_ = 0 denoting a single hidden layer. Feature selection was applied, thereby reducing the number of variables (NV) for each optimized ANN. Accordingly, each candidate solution proposed by the CCLNNA-ANN automatic process was encoded as a 74-component vector comprising: (i) ten integer slots specifying the compound indices assigned to Va/IT subsets; (ii) two integers defining the ANN topology (N_1_ and N_2_); and (iii) 62 variable switches. For the variable block, continuous values in the [0,1] range produced by the optimizer were binarized with a 0.8 cutoff, yielding 1 (variable included) or 0 (excluded).

In contrast to Sagrado et al.^12^—which tuned CCLNNA to optimize recommendation performance—our objective here is to maximize predictive accuracy across the full EES–MP profiles. The CCLNNA objective function (Fobj; eq 5) combines two penalized quality terms, *pQ* (eq 2) and *pE* (eq 4). The *pQ* metric is a modified coefficient of determination that penalizes departures from the ideal regression line in the validation plot (predictions **Y** vs targets **T**) and discourages subset-to-subset instability to mitigate overfitting. Specifically

2 pQ=100·(mQ−pOverfit·sQ)

where *Q* is the subset-specific penalized determination index, *mQ* and *sQ* are the mean and standard deviation of *Q* across the Tr/Va/IT subsets, and pOverfit is a user parameter (fixed at 2 in this study). For each subset, *Q* is calculated according to eq 3

3 $$Q=R_{\mathrm{sign}}^{2}-\left|1-b_{1}\right|-\left|b_{0}\right|$$

with *R*_sign_^2^ = *R* × |*R*|, (here *R* is the Pearson correlation between **Y** and **T** in the validation plot) and *b*_1_, *b*_0_ are the slope and intercept of the same plot. Thus, *Q* is maximized only when the fit is strong and approximately unbiased (*b*_1_ ≈ 1, *b*_0_ ≈ 0).

The complementary penalty metric, pE, counts and weights the absolute prediction errors |**Y**–**T**|, giving higher weights to larger errors and to more critical subsets (IT > Va > Tr). Errors are grouped as Level-1 (0.2 < |Y–T| < 0.4), Level-2 (0.4 ≤ |Y–T| < 0.6), and Level-3 (|Y–T| ≥ 0.6). Tr1, Tr2, and Tr3 are the numbers of training compounds in Levels 1–3; Va1, Va2, and Va3 are the corresponding counts for validation; IT1, IT2, and IT3 for internal test. The metric is

4 pE=0.1·(Tr1+Va1+IT1)+0.2·Tr2+0.3·Tr3+1·Va2+1.5·Va3+2·IT2+3·IT3

The objective function Fobj combines both metrics with equal weighting (*W* = 0.5)

5 Fobj=W·pQ+(1−W)·pE

Higher *F*obj values indicate ANN with a strong overall fit, limited overfitting, and low error—features consistent with better generalization on the current data set. The CCLNNA control parameters, maximum iterations (=500) and population size (=250) were fixed a priori. Under these conditions, we generated a total of 70 optimized ANNs, which we considered sufficient at this proof-of-concept stage to assess the potential of the proposed strategy.

Table S3 provides (i) a detailed description of the algorithmic modifications introduced with respect to the original CCLNNA implementation^29^ and (ii) the key MATLAB code lines used to couple CCLNNA with the standard feed-forward neural network routines (feedforwardnet, train) from the Deep Learning Toolbox (MathWorks), to facilitate reimplementation by readers familiar with MATLAB.

Among the different machine-learning alternatives, we prioritized an optimized ANN–consensus workflow at this proof-of-concept stage, as it is particularly suited to address the expected extreme nonlinearity and multioutput nature of the EES–MP response matrix and to enhance robustness. ANNs are widely recognized as a flexible and robust family of models for tackling such complex, multioutput nonlinear problems. All scripts were written and adapted by the authors using conventional scientific programming, without the use of generative AI tools.

### ANN-Consensus Model

To enhance the reliability and robustness of predictions, an ANN-consensus model was generated by integrating a limited set of the best-performing individual ANNs, selected based on their quality metrics, visual inspection of validation plots and EES-misclassification counts with respect to the EES threshold. The outputs of the ANN-consensus model (**Y** values from individual ANNs) were combined using robust nonparametric statistics, specifically the median (Median­(**Y**)) and the median absolute deviation (MADe), a robust estimator of the standard deviation statistic.^30^

## Results and Discussion

### Prediction of EES-MP Profiles Using the ANN-Consensus Model

From the 70 ANNs generated, we selected the 10 best performers and ranked them based on visual inspection of validation plots. Table S4 summarizes the performance metrics, and Table S5 lists the final solution-vector parameters. As expected, these ANNs differ in the selected Tr/Va/IT splits, architectures, and input variables (Table S5). A clear gap in misclassification separates the top five ANNs from the rest (Table S4); accordingly, we combined these five into an ANN-consensus model by aggregating their **Y**-outputs using the median­(**Y**) and the corresponding MADe as uncertainty estimation, reasoning that an independently optimized ensemble is more reliable and actionable than any single ANN, particularly for moderate-size data sets. Figure 1 contrasts the best individual ANN (Rank 1 in Tables S3 and S4 and Figure 1A) with the consensus (Figure 1B) using validation and residual plots. The single model captures the overall Y–T trend for EES but shows widely dispersed residuals—especially for several validation compounds—consistent with some overfitting. The consensus markedly compresses residual spread, curbs extreme errors, and improves robustness to outliers. Figure 1 confirms that model ensembling smooths ANN-specific errors and yields more consistent, reliable performance than any individual ANN.

**Figure 1:**
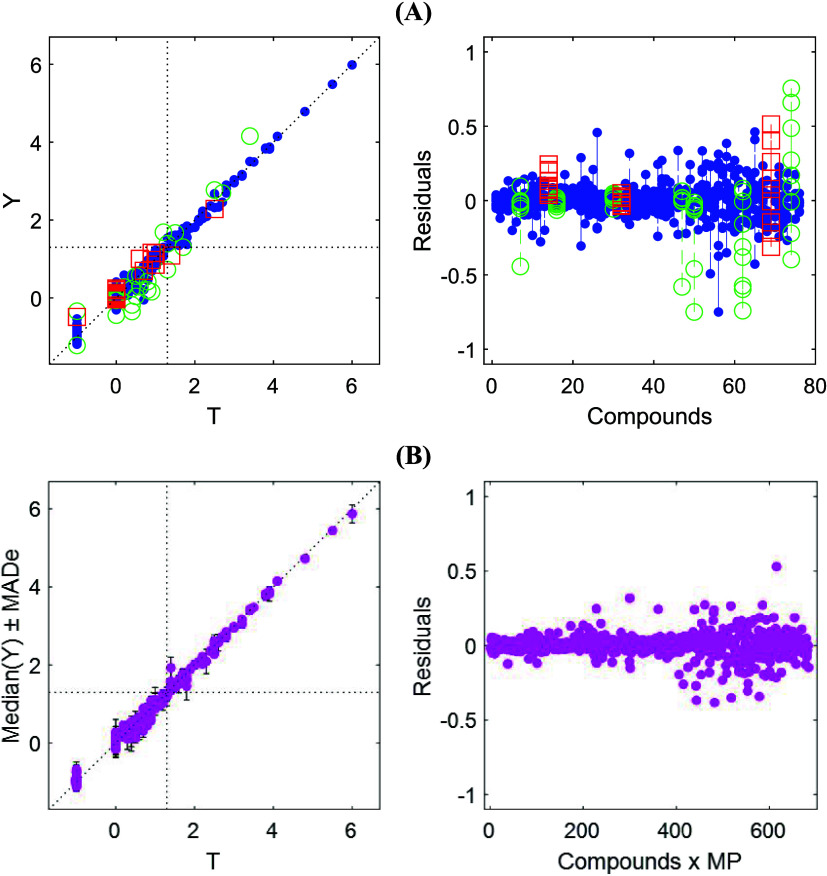
Predicted (**Y**) vs experimental (**T**) EES (descaled) validation plots with residuals plot. (A) Best-performing single ANN (Rank 1, Table S5): training (●), validation (○), and test (□) sets. (B) ANN consensus model (Ranks 1–5, Table S5): Median­(**Y**) prediction (●) with uncertainty expressed as MADe. The dotted line represents the ideal 1:1 relationship; dashed lines indicate the EES threshold (1.3) for acceptable enantioseparation.

Further insight into model performance is provided by comparing experimental and predicted EES-MP profiles across all nine MPs. Figure 2 presents, for all 76 compounds used during the learning stage, experimental profiles (**T-MP**) alongside consensus predictions (Median­(**Y**)-**MP**), with associated uncertainty expressed as MADe.^30^ Compounds with flat profiles (IDs 1–46, with comparable profiles) are grouped for clarity. Predicted profiles closely match experimental trends for most compounds, indicating that the ANN-consensus model effectively captures characteristic EES-MP patterns. The near overlap of Median­(**Y**) and **T** confirms high predictive accuracy, while minimal MADe values reflect excellent precision. The largest deviations were observed for compounds ID 54 (MP 5), ID 58 (MP 5), and ID 69 (MP 3); however, none resulted in misclassification relative to the EES = 1.3 threshold, further supporting the reliability of the consensus approach.

**Figure 2:**
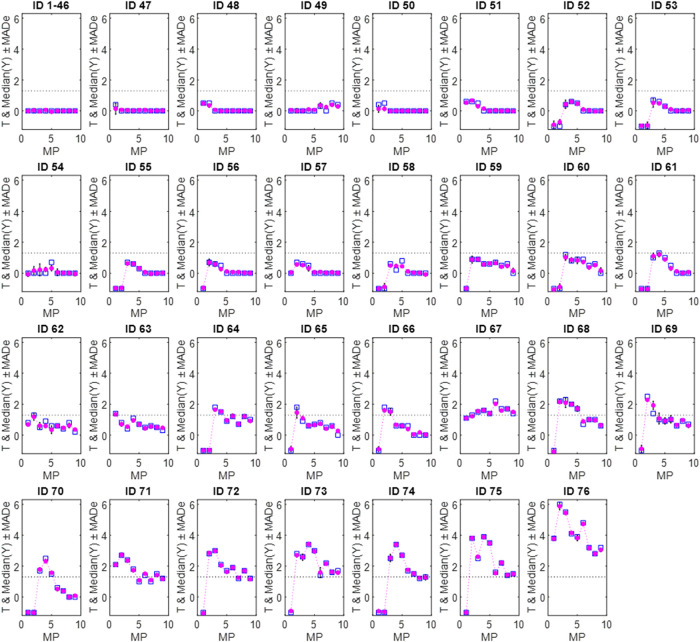
Descaled EES–MP profiles for experimental data (**T**, □) and ANN-consensus predictions (●, Median­(**Y)** ± MADe) for 76 training-stage compounds (IDs in Table S1). The MP index corresponds to distinct mobile-phase compositions (see Experimental Section). Dashed lines indicate the EES decision threshold (1.3) for acceptable enantioseparation.

### Predictive Performance of the ANN-Consensus Model on Unseen Compounds

The current framework was applied prospectively to two external test compounds—fluoxetine (a widely prescribed antidepressant) and lormetazepam (a commonly used hypnotic benzodiazepine)—using only their selected molecular descriptors as inputs. Both compounds were entirely unseen during ANN model building yet fall within chemotypes represented in the training set, thereby providing an additional verification of the model’s practical utility within its applicability domain. Their selection probes model performance on clinically and analytically relevant drugs for which enantioselective analysis is critical for patient safety, therapeutic efficacy, and the implementation of robust health-related analytical workflows.

For each compound, the ANN-consensus generated full EES–MP profiles across nine aqueous–acetonitrile compositions and nominated, a priori, the conditions predicted to yield high EES. Subsequent experiments reproduced the predicted profile trends and confirmed the feasibility calls (EES ≥ 1.3 at the nominated compositions), thereby corroborating practical utility and motivating further development beyond the proof-of-concept stage. Moreover, the ability to propose high-EES mobile-phase compositions and to anticipate lack of enantioresolution from molecular structure broadens the potential of this strategy, specific for Cellulose-1, for mobile-phase selection.

For fluoxetine (Figure 3, left), the model predicted a flat EES-MP profile, with median values close to zero and low uncertainty across all MPs, well below the 1.3 threshold, indicating negligible enantioseparation. In contrast, lormetazepam (Figure 3, right) showed median EES values well above 1.3 for several MPs (notably MP = 3–5), though with higher uncertainty. Among these, MP 4 emerged as the most promising condition, combining a high median EES with relatively low uncertainty, and thus representing the preferred candidate for chromatographic experiments.

**Figure 3:**
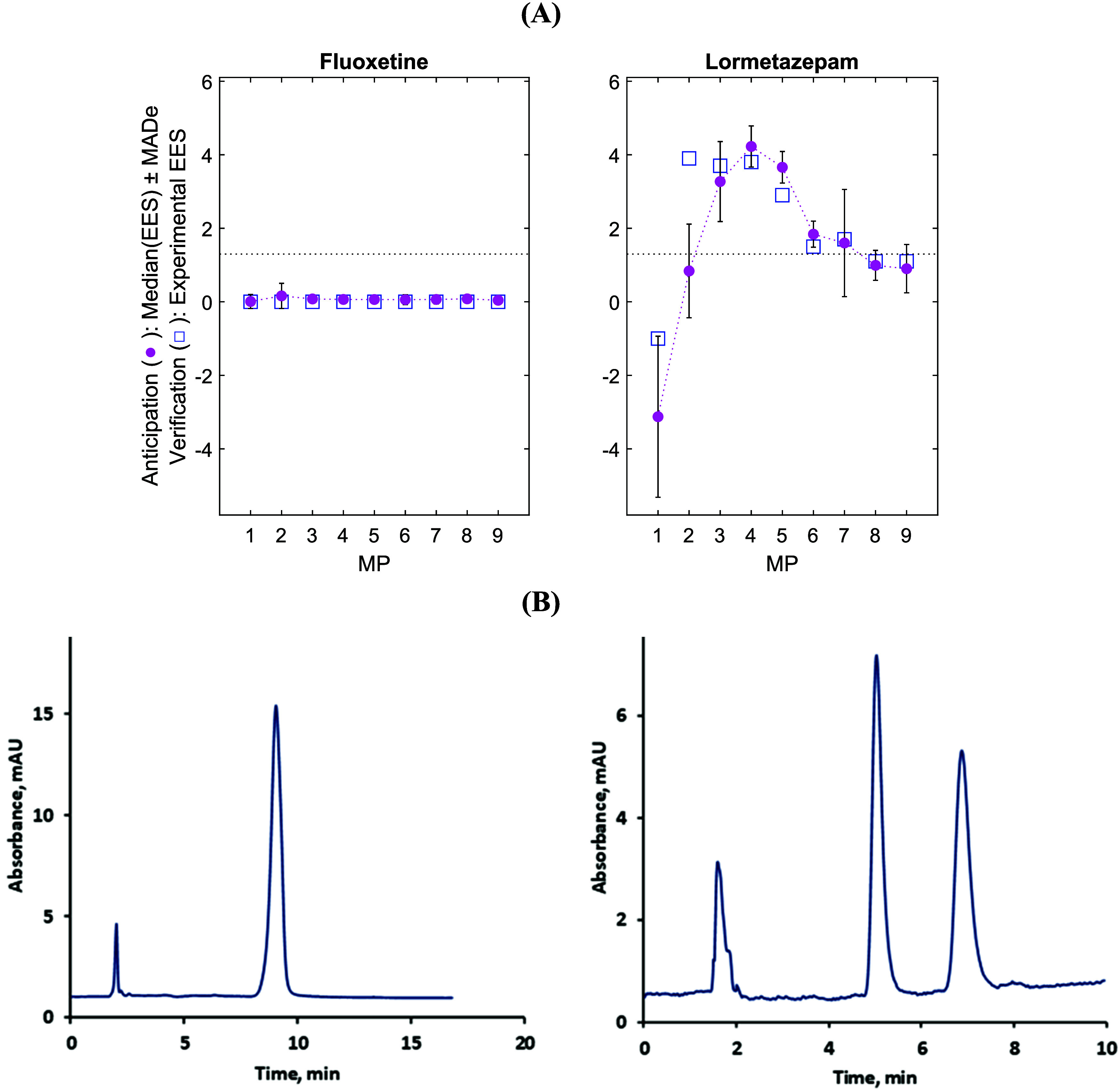
Anticipation and subsequent experimental verification on external compounds: fluoxetine and lormetazepam. (A) Descaled EES–MP profiles: ANN consensus **Y** (●, median ± MADe; anticipation) and **T** (□; verification). (B) Chromatogram verification examples for fluoxetine at MP = 9 and lormetazepam at MP = 4 under ANN-guided conditions. Other information as in Figure 2.

Experimental verification against **T** values (Figure 3) confirmed the suitability of the decisions. Fluoxetine showed no enantioseparation across all MPs, consistent with predictions. For lormetazepam, predictions were accurate for MPs 3–5, particularly MP = 4, while MPs 1–2 showed larger deviations. Importantly, the optimal MP identified by the ANN-consensus model (MP = 4) led to successful enantioseparation, demonstrating that the consensus model can effectively guide MP selection in practice.

The contrast in MADe values—similar to those in the learning stage (Figure 2) for the nonresolved compound but higher for the resolved one (Figure 3)—reflects the class distribution captured by the ANNs. For the compounds studied, non- or poorly resolved cases (EES < 1.3) are more prevalent for Cellulose-1 (see Figure S1), which prioritizes stable modeling of the majority class and maintains prudent, data-consistent predictions for the minority class. Nevertheless, the consensus model provides reliable feasibility calls and smooth EES–MP profiles. This behavior is particularly valuable in pharmaceutical R&D, where rapid and well-calibrated screening enables early deprioritization of unlikely separations and efficient allocation of resources to the most promising mobile-phase compositions. Although the present implementation is a proof of concept limited to a single polysaccharide-based CSP (Cellulose-1) and aqueous–acetonitrile eluents, expanding the training set to additional chiral stationary phases and solvent systems should enhance stability for resolved cases and broaden applicability—ultimately moving toward a robust, generalizable AI-guided platform for chiral method development.

## Conclusions

This work introduces the Efficient Enantioseparation (EES) parameter as a practical metric that integrates resolution and retention factor, providing a realistic assessment of chiral separation performance, and enabling integration into optimization–prediction frameworks such as the CCLNNA–ANN. For the first time, we demonstrate that full EES-MP profiles can be predicted using an AI strategy based on ANN-consensus modeling, which improves robustness and accuracy compared to individual networks and captures characteristic trends across diverse mobile phases.

Testing with external test compounds confirms the potential of this AI strategy as an indirect approach for guiding mobile-phase selection. The consensus model acts as a decision-support tool limiting the reliance on broad trial-and-error experimentation while still requiring routine laboratory confirmation. By establishing a proof-of-concept for data-driven modeling of EES–MP profiles, this predictive framework also advances green analytical chemistry by reducing reagent consumption, experimental workload, and environmental impact.

The results achieved—accurate reproduction of EES–MP profiles, reduced residual dispersion via consensus modeling, and successful prospective predictions—provide a solid foundation for future development. Next steps include (i) data expansion for Cellulose-1 to stabilize EES–MP profiling, leveraging the current model to prescreen and stratify candidates—flagging molecules predicted to be resolved (EES ≥ 1.3) or unresolved (EES < 1.3)—so as to balance the data set across both classes, (ii) staged extension to other polysaccharide CSPs and solvent systems, and (iii) external validation across laboratories, ultimately moving toward practical deployment.

## Supplementary Material





## Notes

Details of compounds used in learning and external test stages; heatmap of EES-MP profiles; list of 62 structural descriptors used for modeling; adapted MATLAB code for the CCLNNA algorithm; hyperparameters and performance metrics for the top 10 artificial neural networks (PDF)
Chromatographic and molecular variables dataset (XLSX)
