# Correction to “Toward AI-Assisted Greener Chiral HPLC: Predicting Efficient Enantioseparation–Mobile Phase (EES–MP) Profiles for MP Selection—A Lux Cellulose‑1 Case Study”

**DOI:** 10.1021/acs.analchem.6c02618

**Published:** 2026-04-28

**Authors:** Carlos Pardo-Cortina, Laura Escuder-Gilabert, María José Medina-Hernández, Salvador Sagrado, Yolanda Martín-Biosca

A typographical error is identified in eq 5 of the published article.

Equation 5 defines the objective function (*F*obj) optimized by the CCLNNA algorithm. This function combines the metrics *pQ* and *pE* to generate artificial neural network (ANN) models with strong overall fit, limited overfitting, and low prediction errors. However, the original formulation incorrectly implies that higher residuals (i.e., higher *pE*) contribute positively to the regression quality, as they are added to the quality metric *pQ*.

The correct expression of eq 5 is

$$F_{obj}=W\cdot pQ-\left(1-W\right)\cdot pE$$ 5

In the corrected form, the regression quality is properly penalized by the residual error term.

This typographical error is confined to the manuscript presentation. The CCLNNA algorithm was implemented using the correct expression; therefore, the reported results and conclusions remain unaffected.

