# Supplementary material for: Toward AI-Assisted Greener Chiral HPLC: Predicting Efficient Enantioseparation–Mobile Phase (EES–MP) Profiles for MP Selection—A Lux Cellulose-1 Case Study
Source: Anal Chem. 2025 Dec 22;98(1):927–33. doi: 10.1021/acs.analchem.5c06117 (PMC12809703; doi:10.1021/acs.analchem.5c06117)
Supplement: Supplementary file 1 [file ac5c06117_si_001.pdf]

SUPPORTING INFORMATION

Toward AI-Assisted Greener Chiral HPLC: Predicting Efficient Enantioseparation–Mobile Phase (EES–MP) Profiles for MP Selection – A Lux Cellulose-1 Case Study.

Carlos Pardo-Cortina[a], Laura Escuder-Gilabert[a], María José Medina-Hernández[a], Salvador Sagrado[a,b*], Yolanda Martín-Biosca[a*]

[a] *Departamento de Química Analítica, Universitat de València E- 46100, Burjassot, Valencia, Spain*

[b] *Instituto Interuniversitario de Investigación de Reconocimiento Molecular y Desarrollo Tecnológico (IDM), Universitat Politècnica de València, Universitat de València, 46100, Burjassot, Valencia, Spain*

*Corresponding author: S. Sagrado (sagrado@uv.es) Phone: 34-963544878

* Corresponding author: Y. Martín-Biosca (Yolanda.martin@uv.es). Phone: 34-963543186

**TABLE OF CONTENTS**	**PAGE**
**Table S1.** Number (ID), family and name of compounds included in learning stage (Tr, Va, IT) and external test compounds	S2
**Figure S1.** Heat map of EES values for the learning compounds	S3
**Table S2.** Structural descriptors used for modelling	S4
**Table S3.** Adaptation of the original CCLNNA MATLAB code	S7
**Tables S4 and S5.** Hyperparameters and metrics for the top 10 ANNs	S9

Table S1.- Number (ID), family and name of compounds included in learning stage (Tr, Va, IT) and external test compounds.

ID	Family [a]	Name	ID	Family [a]	Name
**Learning stage (Tr, Va and IT)**					
1	AAD	Disopyramide[b]	39	AD	Venlafaxine
2	AAD	Mexiletine[b]	40	ADB	Pioglitazone
3	AAD	Propafenone[b]	41	AD	Selegiline
4	AD	Bupropion[b]	42	AF	Nuarmimol
5	AH	Brompheniramine[b]	43	IN	Lufenuron
6	AH	Carbinoxamine[b]	44	AF	Tebuconazole
7	AH	Chlorpheniramine[b]	45	AD	Citalopram[b]
8	AH	Doxylamine[b]	46	BD	Clenbuterol[b]
9	AH	Fexofenadine[b]	47	LA	Prilocaine[b]
10	LA	Bupivacaine[b]	48	AD	Viloxazine[b]
11	LA	Mepivacaine[b]	49	BD	Propranolol[b]
12	LA	Propanocaine[b]	50	BD	Isoprenaline[b]
13	ANP	Bicalutamide[b]	51	PPI	Pantoprazole[b]
14	BB	Acebutolol[b]	52	AH	Chlorcyclizine
15	BB	Atenolol[b]	53	AD	Trimipramine[b]
16	BB	Metoprolo[b]l	54	AH	Cetirizine[b]
17	BD	Salbutamo[b]l	55	CaB	Cilnidipine[b]
18	BB	Timolol[b]	56	CaB	Verapamil[b]
19	BD	Bambuterol[b]	57	PPI	Lansoprazole[b]
20	BD	Orciprenaline[b]	58	AF	Imazalil[b]
21	BD	Terbutaline[b]	59	AF	Penconazole[b]
22	CaB	Felodipine[b]	60	AF	Benalaxyl[b]
23	ACD	Procyclidine[b]	61	AAD	Flavanone
24	APD	Ethopropazine[b]	62	BB	Oxprenolol
25	APD	Promethazine[b]	63	AH	Dimetindene
26	APD	Thioridazine[b]	64	AD	Mianserin[b]
27	AH	Trimeprazine[b]	65	AD	Nomifensine[b]
°28	PPI	Rabeprazole[b]	66	AH	Hydroxyzine[b]
29	IN	Nicotine	67	BB+BD	Pindolol[b]
30	CI	Rivastigmine	68	AH	Orphenadrine[b]
31	AAD	Flecainide	69	AF	Hexaconazole[b]
32	BB	Sotalol	70	AF	Vinclozolin
33	HB	Fenoxaprop	71	ANP	Aminoglutethimide[b]
34	AN	Sibutramine	72	AF	Myclobutanil[b]
35	SSRI	Norfluoxetine	73	BZD	Oxazepam
36	BB	Celiprolol	74	CI	Donepezil
37	PPI	Omeprazole	75	BZD	Lorazepam
38	TLD	Chlorthalidone	76	AF	Metalaxyl[b]
**External test**					
1	AD	Fluoxetine[b]	2	BZD	Lormetazepam

[a] anorectics (AN), antiarrhythmic (AAD), anticholinergics (ACD), antidepressants (AD), antidiabetics (ADB), antifungals/fungicides (AF), antihistamines (AH), antineoplastics (ANP), antipsychotics (APD), β-blockers (BB), bronchodilators (BD), benzodiazepines (BZD), calcium channel blockers (CaB), cholinesterase inhibitors (CI), herbicides (HB), insecticides (IN), local anesthetics (LA), proton pump inhibitors (PPI), selective serotonin reuptake inhibitors (SSRI), and thiazide-type diuretics (TLD).

[b]. Data for these compounds have been reported in a previous study (ref. 27).

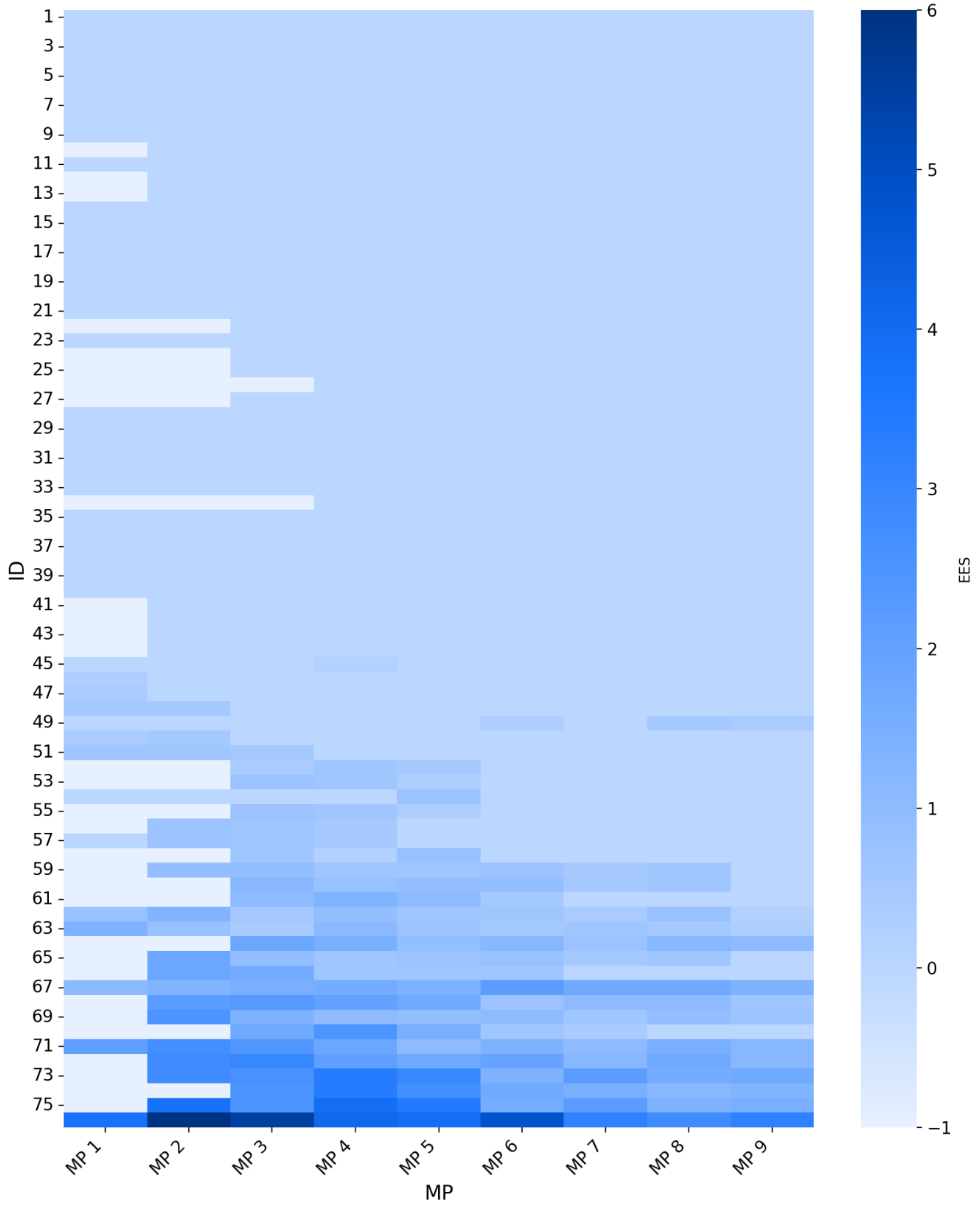

Fig. S1. Heatmap of EES–MP profiles for the 76 learning-stage compounds (Table S1), used as the target **T** matrix after autoscaling the data.

Table S2.- Structural variables used for modelling.

Number	Symbol	Description
x1	C*X	Number of halogen atoms bonded to the chiral atom (C*- halogen)
x2	C*XH	Number of –OH, –NHR or $–NH_2$ groups bonded to the chiral atom
x3	C*hA	Number of aromatic heterocycles groups bonded to the chiral atom (C*-aromatic heterocycles)
x4	C*a	Number of aliphatic groups bonded to the chiral atom (C*-aliphatic)
x5	C*H	Number of hydrogen atoms bonded to the chiral atom (C*-H)
x6	C*C=O	Number of carbonyl groups bonded to the chiral atom: C*- amide ($–C(=O)NR_1R_2$), carbonyl (-C(=O)-R) and ester (–C(=O)-OR) groups
x7	C*A	Number of aromatic groups bonded to the chiral atom (C*-aromatic)
x8	C*XR	Number of –OR, –SR and $–NR_2$ bonded to the chiral atom
x9	Mr	Molecular weight
x10	HBA	**H-bond acceptors:** number of hydrogen bond acceptors in the molecule
x11	HBD	**H-bond donors:** number of hydrogen bond donors in the molecule
x12	PSA	Polar surface area
x13	ST	Surface tension
x14	abc	**Aliphatic bond count:** number of non-aromatic bonds in the molecule (excluding bonds of hydrogen atoms)
x15	Abc	**Aromatic bond count**: number of aromatic bonds in the molecule
x16	bc	**Bond count**: number of bonds in the molecule including hydrogens
x17	Rbc	**Ring bond count**: number of ring bonds
x18	aRc	**Aliphatic ring count**: number of those rings in the molecule, which have non-aromatic bonds
x19	ARc	**Aromatic ring count**: number of aromatic rings in the molecule
x20	cRc	**Carbon ring count**: number of rings in the molecule, which contain carbon atoms only
x21	fARc	**Fused aromatic ring count:** number of aromatic rings having common bonds with other rings
x22	fRc	**Fused ring count**: number of fused rings in the molecule (having common bonds)
x23	HRc	**Hetero ring count**: number of rings in the molecule, which contain hetero atoms

Table S2.- continued.

x24	HaRc	**Heteroaliphatic ring count**: number of aliphatic heterocycles in the molecule
x25	HARc	**Heteroaromatic ring count**: number of aromatic heterocycles in the molecule
x26	Rc	**Ring count:** number of rings in the molecule
x27	RSc	**Ring system count:** number of disjunct ring systems
x28	sRSs	**Smallest ring system size:** number of rings in the smallest ring system
x29	Bi	**Balaban index**: Balaban distance connectivity of the molecule, which is the average distance sum connectivity
x30	fsp3	number of sp^3 hybridized carbons divided by the total carbon count
x31	C	Number of carbon atoms in the molecule
x32	N	Number of nitrogen atoms in the molecule
x33	O	Number of oxygen atoms in the molecule
x34	S	Number of sulfur atoms in the molecule
x35	Cl	Number of chlorine atoms in the molecule
x36	F	Number of fluorine atoms in the molecule
x37	CN	Number of –CN groups in the molecule
x38	OH	Number of –OH groups in the molecule
x39	NHR	Number of -NHR groups in the molecule
x40	NR2	Number of-NR_2 groups in the molecule
x41	ROR	Number of R-O-R groups in the molecule
x42	tB	Number of tert-butil groups in the molecule
x43	iP	Number of iso-propyl groups in the molecule
x44	ACH3	Number of -CH_3 groups bonded to an aromatic ring in the molecule
x45	ACl	Number of Cl atoms bonded to an aromatic ring in the molecule
x46	AOH	Number of -OH groups bonded to an aromatic ring in the molecule
x47	AOR	Number of –OR groups bonded to an aromatic ring in the molecule
x48	ACOOR	Number of -COOR groups bonded to an aromatic ring in the molecule
x49	ANH2	Number of –NH_2 groups bonded to an aromatic ring in the molecule
x50	AF	Number of -F atoms bonded to an aromatic ring in the molecule
x51	ACF3	Number of -CF_3 groups bonded to an aromatic ring in the molecule
x52	ANHCOR	Number of - NHCO-R groups bonded to an aromatic ring in the molecule

Table S2.- continued.

x53	ACA	Number of moieties Ar-C-Ar
x54	A12	Number of aromatic groups with 1,2 substitutions.
x55	A14	Number of aromatic groups with 1,4 substitutions
x56	A123	Number of aromatic groups with 1,2,3 substitution
x57	A124	Number of aromatic groups with 1,2,4 substitution
x58	NA	Number of nitrogen aromatic groups
x59	NRC	Number of tertiary amines in aliphatic cycles
x60	SC	Number of S atoms in aliphatic cycles
x61	logP	Logarithm of the partition coefficient
x62	logD	log *P* at working pH

Table S3. Adaptation of the original CCLNNA (Copyright (c) 2021, yiying zhang. All rights reserved) MATLAB code (ref. 26)

Lines/script of the original code	Adapted code (marked new/modified variables)	Comments
1 [BestCost,BestValue]=CCLNNA(fhd,nPop,nVar,VarMin,VarMax,MaxIt,X)	[BestCost,BestValue,Xopt]=CCLNNA(fhd,nPop,nVar,VarMin,VarMax,MaxIt,X)	1 and 3
41 cost(i)=fhd(x_pattern(i,:));	Delete this line.	
44 [COST,index]=min(cost);	Delete line 44 and replace it with:<br>x_pattern(:,1:nVaIT+nArquit)=round(x_pattern(:,1:nVaIT+nArquit));<br>x_pattern(:,nVaIT +nArquit+1:end) = …<br>x_pattern(:,nVaIT +nArquit+1:end)>CritVarXon;<br>for i=1:nPop<br>[Fobj,pQ,pE] = fhd(x_pattern(i,:), W);<br>cost(i)=Fobj;<br>end	2
69-173<br>Main loop (for ii==2:Max It)	Each time x_pattern is updated insert:<br>x_pattern(:,1:nVaIT+nArquit)=round(x_pattern(:,1:nVaIT+nArquit));<br>x_pattern(:,nVaIT +nArquit+1:end) = …<br>x_pattern(:,nVaIT +nArquit+1:end)>CritVarXon;<br><br>Apply the same procedure to the variable XTarget.<br><br>Replace any line: cost(i)=fhd(x_pattern(i,:)); with the loopt:<br>for i=1:nPop<br>[Fobj,pQ,pE] = fhd(x_pattern(i,:), W);<br>cost(i)=Fobj;<br>end	2
170	Insert:<br>Xopt=XTarget;	1

1) CCLNNA function, adapted to (i) return Xopt (the final optimized **solution**) and (ii) call fhd as the problem-dependent objective function, defined in terms of ANN outputs and their associated metrics (see 3).

2) **Solution** values (x_pattern) are rounded to integers for the Va/IT compound indices (nVaIT) and the architecture parameters (nArquit); **X**-variable entries are set to 1 only when they exceed the CritVarXon threshold.

3) CCLNNA_ANN driver code developed in this work (key MATLAB lines, analogous to the "demo.m" example in ref. 29 to couple CCLNNA with ANN):

MATLAB code key lines (problem-dependent variables are marked).	Explanation
escx=2; escy=2;<br>Max It=500;<br>nPop=250;<br>CritVarXon=0.8;<br><br>pOverfit=2;<br>W=0.5;	Defines autoscaling for **X** and **T** data, respectively.<br>Sets the maximum number of iterations (MaxIt) and the population size (nPop).<br><br>CritVarXon: threshold used to decide whether a variable is selected during optimization.<br>(See Eq. 2)<br>(See Eq. 5)
CL=[<br>1 2 3 4 5 6 7 NaN<br>8 9 10 11 12 13 14 15<br>16 17 18 19 20 21 22 23<br>24 25 26 27 28 29 30 31<br>32 33 34 35 36 37 38 39<br>40 41 42 43 44 45 46 47<br>48 49 50 51 52 53 54 55<br>56 57 58 59 60 61 62 NaN<br>63 64 65 66 67 68 69 NaN<br>70 71 72 73 74 75 76 NaN<br>];<br><br>IndVa=[1 3 4 6 7 8 10]; IndIT=[2 5 9];<br><br>IndN1=1:30; IndN2=0:30;<br><br>nVar=nVaIT+nArquit+NvX;<br><br>VarMin = [min(CL,[],2,'omitnan')' min(IndN1) min(IndN2) zeros(1,NvX)];<br>VarMax = [max(CL,[],2,'omitnan')' max(IndN1) max(IndN2) ones(1,NvX)];	**CL** matrix (10 rows) containing compound indices ordered as in T, where compounds are sorted by increasing EES; selecting one index per row with CCLNNA therefore distributes the chosen compounds across the EES range.<br><br>Row indices in CL from which CCLNNA selects the validation (IndVa) and internal-test (IndIT) compounds (one per row) in each solution.<br><br>Allowed ranges for the number of neurons in the first (N_1) and second (N_2) hidden layers.<br>nVar: length of the solution vector, composed of nVaIT (7 + 3 compound indices for Va and IT), nArquit (2 hidden-layer sizes), and NvX (62 **X**-variables).<br>Lower and upper bounds for each component of the solution vector.
fhd = @ANNfunction;<br><br>rng('shuffle')<br>for i=1:nPop<br>x(i,:)=VarMin +rand(1,nVar).*(VarMax - VarMin);<br>end<br><br>[BestCost,BestValue,Xopt]=CCLNNA(fhd,nPop,nVar,VarMin,VarMax,MaxIt,x)<br><br>selXVar=find(Xopt((nVaIT+nArquit)+1:length(Xopt))==1);	Problem-dependent objective function (ANNfunction; analogous to the "Sphere.m" example in [29] but adapted to ANN-based prediction).<br>Randomizes the starting solutions across runs.<br>Initial population of random **solutions**.<br><br>See comment 1 (adapted CCLNNA header).<br><br>Indices of **X**-variables selected at the end of the optimization (feature selection).

function [Fobj,pQ,pE] = ANNfunction(x, W)<br><br>rng(1)	MATLAB function (using feedforwardnet.m and train.m functions and demos from MathWorks®) that, after training each ANN under the solution encoded by x , computes **Y** predictions and evaluates the metrics from **T** and **Y** (Eqs. 2, 4, and 5).<br><br>It is set immediately before ANN training so that weight initialization and training are deterministic and reproducible, ensuring that all candidate solutions are evaluated under identical ANN stochastic conditions.
function [BestCost,BestValue,Xopt]=CCLNNA(fhd,nPop,nVar,VarMin,VarMax,MaxIt,x);	MATLAB function (adapted version of "CCLNNA.m" from [29]; see comment 1).

Table S4.- Hyperparameters and number of selected variables (NV) for the top 10 ANNs generated by the CLLNNA-ANN process.

**Rank**	**Validation compounds (ID)**	**Internal test compounds (ID)**	**N_1**	**N_2**	**NV**
1	7, 16, 30, 47, 50, 62, 74	14, 32, 69	14	9	23
2	7, 19, 24, 43, 51, 62, 76	15, 32, 68	11	8	13
3	6, 22, 27, 46, 55, 59, 75	14, 33, 69	24	9	14
4	5, 18, 24, 47, 54, 60, 76	14, 36, 69	29	8	23
5	7, 23, 24, 42, 50, 58, 73	15, 39, 69	30	16	22
6	4, 23, 24, 40, 48, 56, 76	14, 32, 69	6	11	23
7	7, 20, 27, 40, 48, 56, 73	14, 32, 69	22	9	12
8	7, 17, 30, 4, 48, 59, 75	8, 37, 69	30	3	22
9	5, 20, 26, 41, 48, 57, 75	8, 32, 69	9	8	9
10	2, 17, 25, 41, 50, 56, 75	15, 39, 69	29	6	17

Table S4 summarizes the hyperparameters associated with the solution vector—Va/IT (validation compounds), network hidden architecture, and number of selected variables (NV)—for the top-10–ranked ANNs. All models included a second hidden layer ($N_2 > 0$), consistent with the high complexity of the $\mathbf{T} = f(\mathbf{X})$ relationship. In several cases, the neuron counts reached the predefined maximum (30 per layer). Deeper networks might capture this complexity more fully, but at the cost of a higher probability of overfitting. Variable reduction from the initial 62 descriptors ranges from 62.9% to 85.5%.

Table S5. Primary metrics for the top 10 ANNs (Table S3). The first five ANNs (shaded in light green) formed the consensus model.

**Rank**	**pQ**	**pE**	**R^2 (%)**	**R^2_{Tr} (%)**	**R^2_{Va} (%)**	**R^2_{IT} (%)**	**Dif**	**Sum**
1	61	29	97	98	90	94	0	2
2	61	48	94	97	89	92	0	2
3	61	37	97	99	86	88	3	3
4	67	39	96	99	91	95	1	1
5	54	28	96	99	81	90	1	3
6	75	40	93	95	88	89	4	4
7	74	41	94	95	92	80	4	10
8	62	40	94	96	83	95	2	12
9	82	53	90	92	82	88	10	12
10	78	43	92	94	84	93	10	16

Dif is the absolute difference between the “up” and “down” **Y** vs. **T** misclassification counts with respect to the EES = 1.3 threshold, indicating potential bias. **Sum** is the total number of misclassifications.

Table S5 reports the primary metrics for the top 10 ANNs (in Table S3). Overall R^2 and R^2_{Tr} values were consistently high (≥ 94%), while R^2_{Va} and R^2_{IT} ranged from 81 to 91 % and 88 to 95 %, respectively, indicating residual overfitting. These results are acceptable but could be improved by combining Y-outputs from several ANNs via a consensus model. Notably, the **Dif** and **Sum** metrics were lower for the top five models, indicating better classification accuracy.
